# Enkephalin-encoding herpes simplex virus-1 decreases inflammation and hotplate sensitivity in a chronic pancreatitis model

**DOI:** 10.1186/1744-8069-4-8

**Published:** 2008-02-28

**Authors:** Hong Yang, Terry A McNearney, Rong Chu, Ying Lu, Yong Ren, David C Yeomans, Steven P Wilson, Karin N Westlund

**Affiliations:** 1 Dept of Neuroscience and Cell Biology, University of Texas Medical Branch, Galveston, TX, USA; 2 Dept of Internal Medicine and Microbiology & Immunology, University of Texas Medical Branch, Galveston, TX, USA; 3 Department of Anesthesia, Stanford University, Stanford, CA, USA; 4 Department of Pharmacology, Physiology, and Neuroscience, University of South Carolina School of Medicine, Columbia, SC, USA; 5 Dept. of Physiology, Chandler Medical Center, University of Kentucky, Lexington, KY, USA

## Abstract

**Background:**

A chronic pancreatitis model was developed in young male Lewis rats fed a high-fat and alcohol liquid diet beginning at three weeks. The model was used to assess time course and efficacy of a replication defective herpes simplex virus type 1 vector construct delivering human cDNA encoding preproenkephalin (HSV-ENK).

**Results:**

Most surprising was the relative lack of inflammation and tissue disruption after HSV-ENK treatment compared to the histopathology consistent with pancreatitis (inflammatory cell infiltration, edema, acinar cell hypertrophy, fibrosis) present as a result of the high-fat and alcohol diet in controls. The HSV-ENK vector delivered to the pancreatic surface at week 3 reversed pancreatitis-associated hotplate hypersensitive responses for 4–6 weeks, while control virus encoding β-galactosidase cDNA (HSV-β-gal) had no effect. Increased Fos expression seen bilaterally in pain processing regions in control animals with pancreatitis was absent in HSV-ENK-treated animals. Increased met-enkephalin staining was evident in pancreas and lower thoracic spinal cord laminae I–II in the HSV-ENK-treated rats.

**Conclusion:**

Thus, clear evidence is provided that site specific HSV-mediated transgene delivery of human cDNA encoding preproenkephalin ameliorates pancreatic inflammation and significantly reduces hypersensitive hotplate responses for an extended time consistent with HSV mediated overexpression, without tolerance or evidence of other opiate related side effects.

## Background

Chronic pancreatic pain has significant negative impact on patient quality of life and mortality. Over 50% of patients with idiopathic or alcoholic pancreatitis report chronic severe pain, responsive initially to morphine or synthetic opioids, but subject to development of tolerance to opiates, addiction and other intolerable side effects. More effective, site-specific management of pain from chronic pancreatitis would significantly impact patients' pain levels, physical functioning, quality of life, general health and survival given the high rate of suicide in patients with this condition. The typical high fat diet of the American public may contribute to even higher rates of pancreatitis, particularly when combined with alcohol.

While alcohol ingestion has been used to induce animal models of pancreatitis, reliable chronic pancreatitis models for translational studies are not available [1,2]. The data produced in many current alcohol-fed pancreatitis models is reported to be variable, and the models are costly and time consuming to produce (~8 months). For this study, we used 40-day-old Lewis rats fed a commercial high fat liquid diet with 6% alcohol. The combined high-fat and alcohol diet given to younger animals greatly accelerated the induction of pancreatitis and allowed persistence through the end of the study at 10 weeks. Hypersensitive thermal responses developed in three weeks and remained through ten weeks allowing study of the HSV overexpression time course.

An explosion of interest in gene therapeutic approaches is occurring as investigators assess the potential of molecular targeting in a variety of clinical treatment approaches, such as for cancer chemotherapy and nervous tissue repair [3-5]. While both viral and non-viral approaches are under study, viral oncolytic therapy (both alone and in combination with conventional therapy) has been found as safe, well tolerated, highly effective and efficient in clinical trials as in animal studies. HSV-1, the common pathogen causing cold sores, is a class of double-stranded DNA viruses with affinity for primary sensory neurons as do all viruses of this class, including herpes zoster (HHV-3) causing severe shingles pain. Both attenuated and replication-competent HSV have been used successfully in over 20 published Phase I/II clinical trials to date for prostate cancer, glioblastoma, melanoma, leukemia and other hematological malignancies [6-9]. Genetically engineered replication-competent HSV-1 oncolytic transgene has been safely delivered to patients who had failed prior therapy. This includes i.v. delivery to patients with hepatic colorectal metastases refractory to the first-line chemotherapy [9] and directly into tumors of the skin, breast, head, neck, and gastrointestinal tract [10]. Histological evidence of tumor shrinkage and necrosis is provided [10]. Adenoviral mediated transfer of the IL-4 gene has also been used to modify inflammatory cell invasion in a chemically-induced model of pancreatitis [11].

While retroviral (lentivirus), adenoviral, adeno-associated and herpes simplex viral vectors are currently under study, advantages for use of herpes viral vectors have been detailed [4,6,12-17]. Since minimal activation of immune responses occurs to initial or prior HSV infections, severe medical illness in immune-competent adults is rarely seen as with other viral vectors, and anti-viral agents are available. Another advantage is that HSV-1 does not integrate into the host genome, as do retroviral and adeno-associated viruses, so insertional mutagenesis is not a concern. It efficiently delivers circular, non-replicating DNA extrachromosomally to the cell nucleus. Importantly, the large HSV-1 cassette can easily be genetically engineered to accommodate multiple transgene modules of any size up to 150 kbp, including foreign DNA, cDNA, and even large transcriptional regulatory sequences to provide cell type specificity. Typically, the vector is loaded with CMV promoter which amplifies its expression efficiency. HSV-1 can be rendered (1) replication conditional for added control, (2) replication defective so it cannot produce viral proteins required for productive infection or (3) replication-competent to prolong expression and perhaps even allow advantageous reactivation. Utilizing the cell's own machinery to "minipump" desired therapeutic agents, HSV vectors can efficiently and effectively amplify production of selected gene product(s) persisting on the order of weeks-months in non-replicating neurons. Thus, the HSV-1 transgene provides innumerable possibilities for modification and customization to improve gene delivery.

Our research goal was to assess the impact of met-enkephalin overexpression in the high-fat and alcohol-induced pancreatitis model using a replication defective HSV vector to deliver human cDNA encoding preproenkephalin. This same overexpression vector has been used for uptake by primary afferent nociceptor endings via a cutaneous route, resulting in diminution of nociceptive behaviors in other models of inflammatory injury [13,18,19]. In the current project, HSV-1 viral vectors are applied directly to communicating sensory neurons in inflamed pancreas after development of a hypersensitive behavioral state induced with a high-fat and alcohol diet in rats. Impressive abrogation of pancreatic inflammation and hotplate hypersensitivity was detected in animals receiving the HSV-ENK vector. Accompanying these findings were increased tissue levels of met-enkephalin in thoracic spinal cord and pancreas (but not in cervical spinal cord, liver, bladder or colon), and decreased Fos expression in spinal cord and midbrain. These findings support previous assertions that gene therapeutic approaches are useful clinical tools for extended treatment of chronic inflammatory conditions [12,16,20], and indicate a key role for met-enkephalin overexpression in tissue repair/protection. Direct local use of HSV viral vectors clinically would specifically avoid central nervous system encroachment and the many unwanted side-effects of opiate use, including tolerance.

## Results

### Pancreas of HSV-ENK-treated rats has normal histopathology

**Figure 1:**
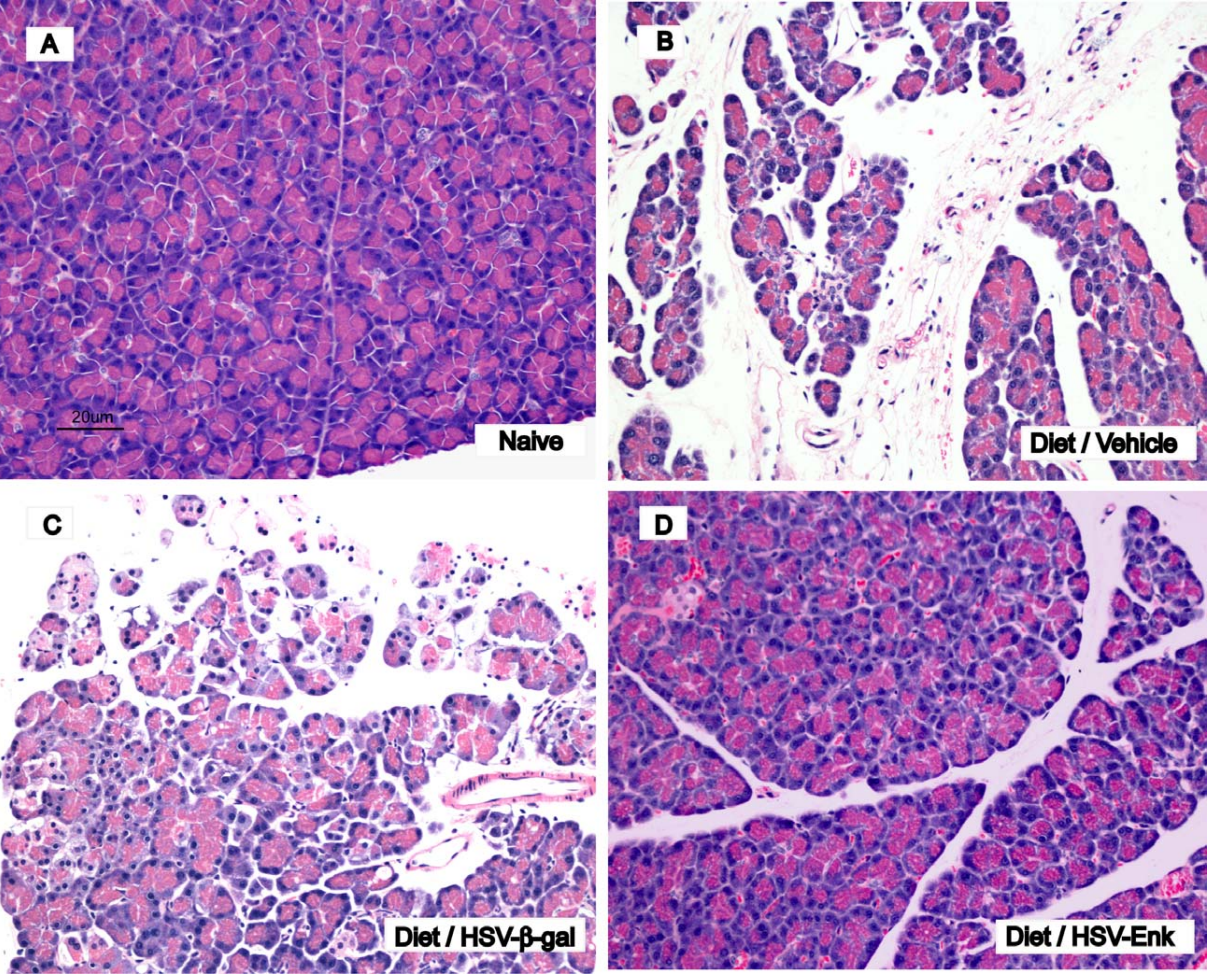
**Histopathology of rat pancreas at week 10**. **A**. Naïve animals were fed low soy chow and given no treatment. **B**. Method control animals given the alcohol and high-fat diet to induce pancreatitis were given pancreatic injection of vehicle (DMEM) only. **C**. Some animals with alcohol and high-fat diet induced pancreatitis were given pancreatic HSV-β-gal applications, serving as the vector control. Note the steatosis, inflammatory cell infiltration, acinar cell necrosis, tissue edema, ductal widening and periductal fibrosis seen in the controls, with alcohol and high-fat diet induced pancreatitis given vehicle or the HSV-β-gal applications (**B **and **C**). **D**. Greatly reduced inflammatory cell infiltration and preservation of pancreatic tissue architecture was seen in animals fed the alcohol and high-fat diet but treated with the HSV-ENK vector. The histopathology of the HSV-ENK vector-treated animals was similar to that of the naïve animals (**A**). Hemotoxylin and eosin (H&E) stain.

Histological examination of pancreata at ten weeks in rats fed with high fat and alcohol diets and treated with vehicle (Fig. 1B) or HSV-β-gal (Fig. 1C) demonstrated tissue edema, steatosis, inflammatory cell infiltration, fibrosis, and acinar necrosis in all rats from these two groups. However, the pancreata of rats fed with alcohol and high fat diet but treated with HSV-ENK (Fig. 1D) showed reduced inflammatory cell infiltration and preservation of pancreatic tissue architecture. As expected, no abnormal acinar architecture was detected in the pancreas of naïve control rats (Fig. 1A). Weight gain monitored weekly was within 15–20% of littermates fed conventional lab chow in accordance with IACUC regulations. Enzymes (amylase and lipase), alcohol and glucose were measured in the blood at the end of the ten week study. No differences were found among the treatment groups. Blood alcohol levels were 120.6 ± 35.4 mg/dL for high fat/alcohol treatment groups compared to 4.75 ± 3.4 mg/dL for naïve rats.

### HSV-ENK treatment decreases hotplate sensitivity in pancreatitis

**Figure 2:**
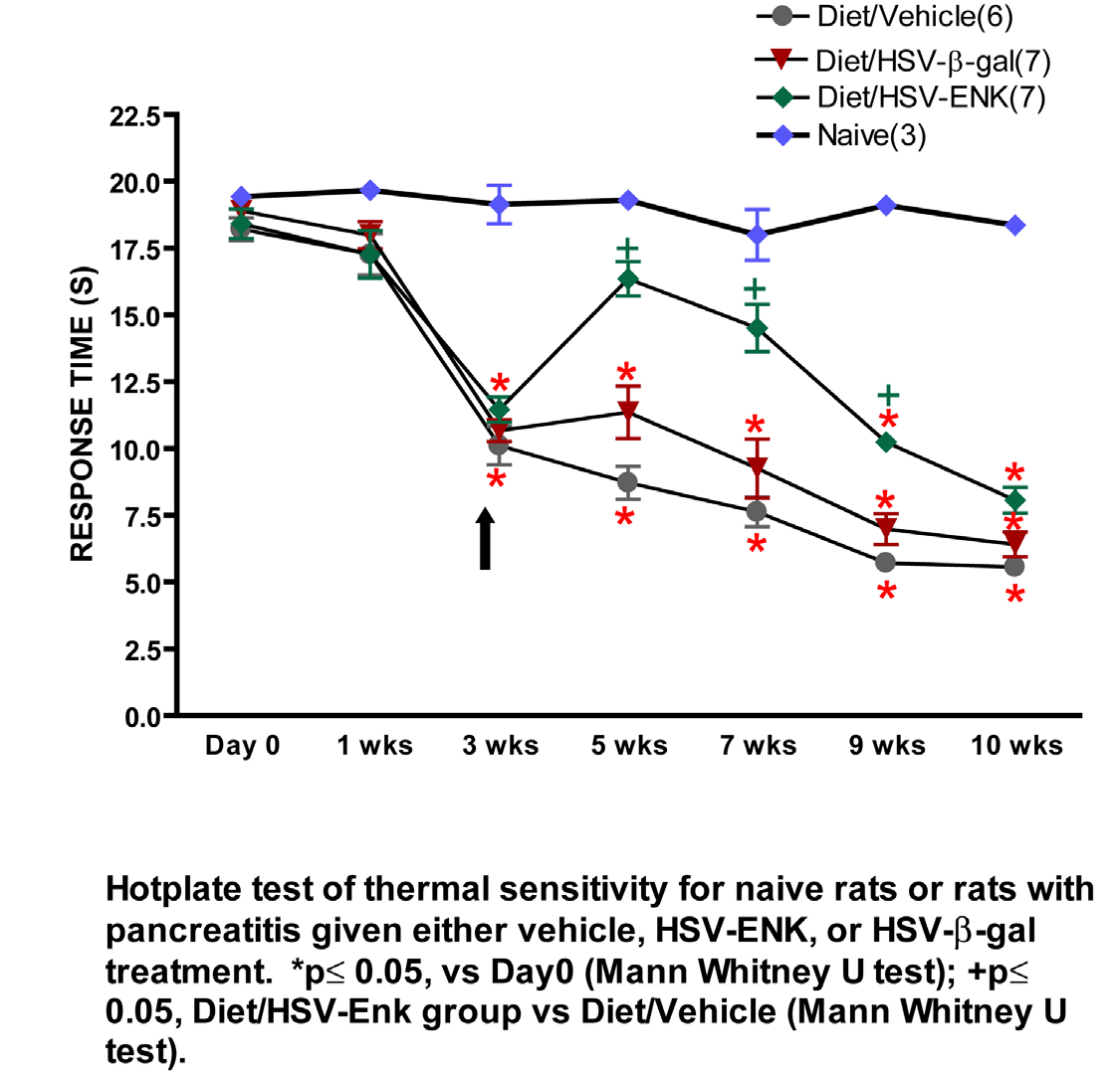
**Hot plate response latency nociceptive behavior measurements**. Hot plate response latency measurements are shown for naïve animals and animal groups with alcohol and high-fat induced pancreatitis. Hot plate test was conducted at baseline before induction of pancreatitis and for ten weeks subsequently. Note the significant shortening of hot plate response latencies for rats on the high fat and alcohol diet after week 3, indicating sensitization. The HSV-ENK treatment (arrow) significantly abrogated the shift in response latency for at least four weeks. Four weeks is typical of HSV vector expression.

Figure 2 demonstrates hot plate response latencies in rats fed the high fat and alcohol diet over the course of the study. Significant decreases in latency thresholds were noted in all groups fed the high fat and alcohol diet at the beginning of the third week indicating development of hypersensitivity. At this time point, the viral vectors and vehicle were applied to the pancreatic surface. In week 5, a significant recovery of withdrawal latencies to near normal values was recorded for animals treated with HSV-ENK (n = 7), compared to animals treated with vehicle (n = 6) or HSV-β-gal vector (n = 7). The analgesic effect of HSV-ENK vector treatment lasted at least 4 weeks (week 5–9) without the apparent development of tolerance. By week 10, all groups fed the diet displayed significantly shortened withdrawal responses compared to naïve rats, indicating thermal hypersensitivity and decreased effect of the met-enkephalin overexpression. Concurrent footpad testing of thermal and mechanical sensitivity with Hargreaves and von Frey tests were negative throughout the ten week study indicating higher order rather than spinal reflexive response changes for this model.

### Increased met-Enkephalin (met-ENK) expression in spinal cord, DRG and pancreas in HSV-ENK-treated animals

The animals used for behavioral studies were sacrificed for immunofluorescent localization studies at the end of week 10. Naïve animals served as untreated controls without pancreatitis. Spinal cord (T7–12), DRG (T7–9) and pancreas tissues were examined for met-ENK, β-gal, and HSV-1 immunoreactivity.

**Figure 3:**
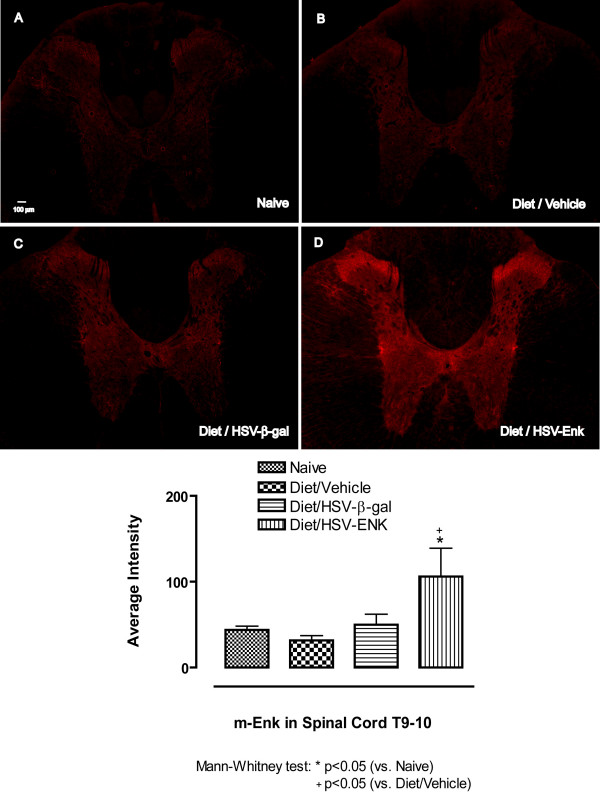
**met-Enkephalin immunohistochemical staining in spinal cord (T9–10)**. **A**. The spinal cord from a naïve rat is shown for comparison to (**B**) the spinal cord of animals with diet-induced pancreatitis at week 10. **C**. The expression of met-ENK after application of vehicle or HSV-β-gal is similar to naïve rats (**A**). **D**. Met-ENK expression was significantly increased in HSV-ENK vector-treated animals compared to controls. Met-ENK in the dorsal horn (laminae I–II) of the thoracic spinal cord was increased bilaterally.

Immunostaining for met-ENK in the spinal cord of HSV-ENK-treated animals at week 10 (Fig 3D, n = 4, 106.1 ± 32.9 arbitrary units) revealed increased densities in Laminae I and II compared to that of HSV-β-gal-treated (Fig 3C, n = 4, 50 ± 12.1 arbitrary units), vehicle-treated (Fig 3B, n = 4, 31.7 ± 5.6 arbitrary units), or naïve animals (Fig 3A, n = 3, 43.7 ± 4.4 arbitrary units). Quantification of the staining confirmed a significant increase in met-enkephalin stain density in HSV-ENK-treated animals (Fig 3E).

**Figure 4:**
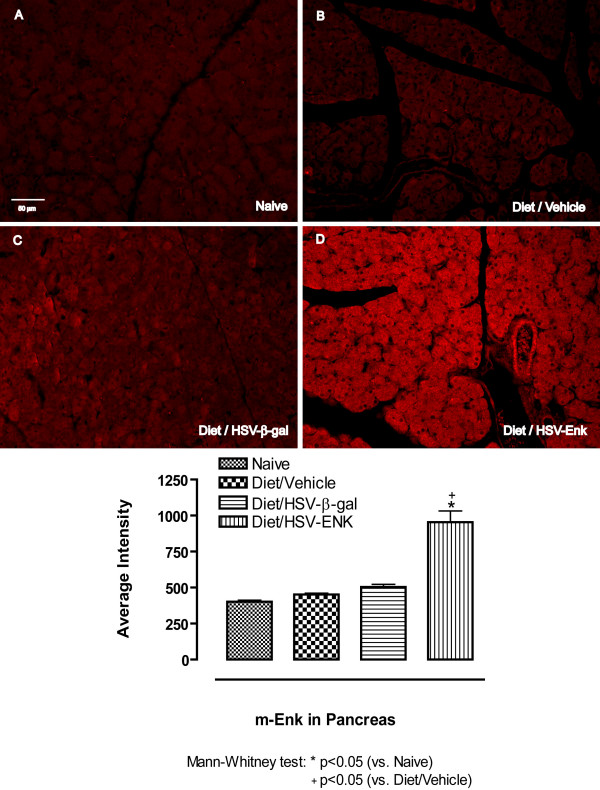
**met-Enkephalin immunohistochemical staining in pancreas**. Photomicrographs and quantification of immunohistochemical staining for met-enkephalin in pancreas are shown for week 10. Minimal or no staining is seen in pancreas of (**A**) naïve and (**B-C**) control animals with alcohol and high-fat diet induced pancreatitis. **D**. Met-ENK expression was significantly increased in the pancreas of HSV-ENK-treated animals fed the same diet compared to the controls.

Staining for met-ENK in pancreata at week 10 revealed significantly increased densities in HSV-ENK-treated animals (Fig 4D, 954.6 ± 77.6 arbitrary units) compared to vehicle or HSV-β-gal-treated animals with pancreatitis (Fig 4B and 4C, 452.2 ± 9.6 and 504.5 ± 18.2 arbitrary units, respectively), or the naïve animals (Fig 4A) (402.1 ± 10.7 arbitrary units). Staining intensities for met-ENK were higher in all animals with pancreatitis compared to naïve animals, particularly those receiving the viral vector. The localization pattern for the met-enkephalin in pancreas was diffuse and appeared granular within the acinar cells, but localization in neuronal terminals and transversely cut axons could not be ruled out.

**Figure 5:**
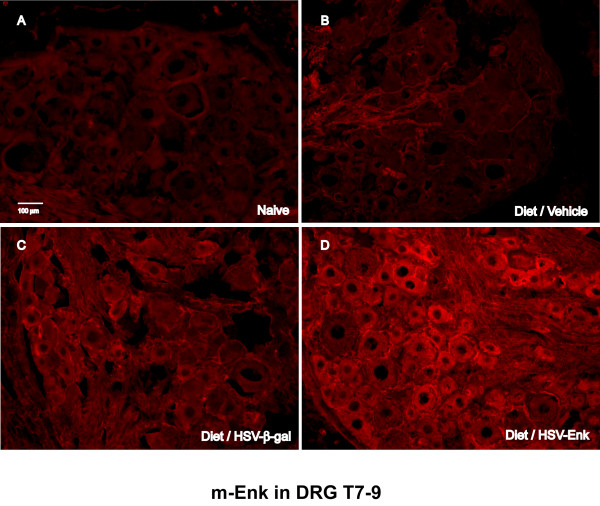
**met-Enkephalin immunohistochemical staining in thoracic DRG**. Photomicrographs of immunohistochemical staining for met-enkephalin in DRG (T7–9) of (**A**) naïve and control animals (**B**, **C**) with alcohol and high-fat diet induced pancreatitis at week 10. Minimal staining is typical of peptides in the absence of axonal transport inhibitors since they are rapidly transported to axonal terminals. **D**. Note the increased met-ENK staining in DRG of animals with HSV-ENK treatment fed the alcohol and high-fat diet, compared to the naïve and control animals. Intracellular staining appears primarily in small and medium sized cells of the T7–T9 DRG of the HSV-ENK treated animals.

Staining for met-ENK is observed in most DRG at week 10 (Fig 5). Higher levels of immunostaining were observed in thoracic DRG of HSV-ENK-treated animals (Fig 5D) compared to vehicle- (Fig 5B) or HSV-β-gal-treated (Fig. 5C) animals with pancreatitis, or the naïve animals (Fig 5A). Staining for β-galactosidase was only detected in the cells of the thoracic DRG from the HSV-β-gal-treated rats with pancreatitis (not shown).

### HSV-1 Protein staining is expressed in DRG of HSV-ENK- and HSV-β-gal-treated animals

**Figure 6:**
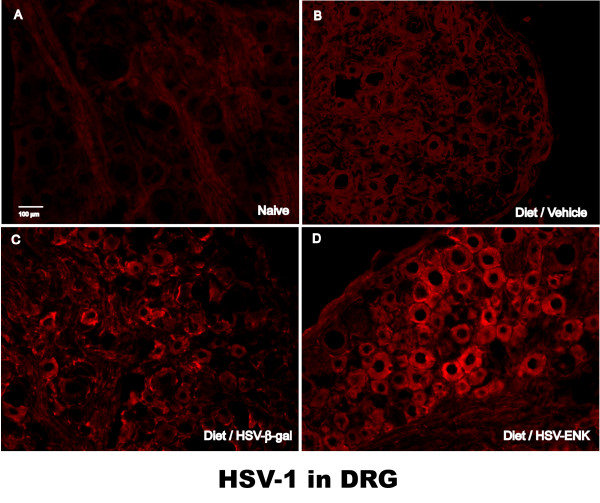
**HSV-1 immunohistochemical staining in thoracic DRG**. Photomicrographs of immunohistochemical staining for human HSV-1 protein at week 10. No stain is evident in dorsal root ganglia of (**A**) naïve animals or (**B**) vehicle-treated animals fed the alcohol and high-fat diet. Note the presence of staining for human HSV-1 protein in DRG of animals given the (**C**) HSV-β-gal and (**D**) HSV-ENK vector treatments. Cytoplasmic localization is noted for HSV in DRG from vector treated animals only in lower thoracic ganglia (T7–T9).

Staining for HSV-1 proteins in the thoracic DRG was observed in many cells in the HSV-ENK- and HSV-β-gal-treated animals in week 10, primarily in the sections illustrated in Figure 6 (Fig 6D and 6C, respectively). As expected, vehicle-treated rats with pancreatitis (Fig 6B) or naïve animals (Fig 6A) had negligible staining of HSV-1 proteins in the thoracic DRG. There was negligible background staining in the cervical DRG of the HSV-ENK- and HSV-β-gal-treated animals (not shown). No immunostaining was detected for HSV-1 proteins in the thoracic spinal cords from any of the groups of animals (not shown). No HSV-1 proteins were detected by immunohistochemistry in pancreas [see Additional file 1], liver [see Additional file 2], duodenum, bladder or colon from any of the treatment groups or naïve animals.

### Decreased Fos expression in brains of HSV-ENK-treated animals

**Figure 7:**
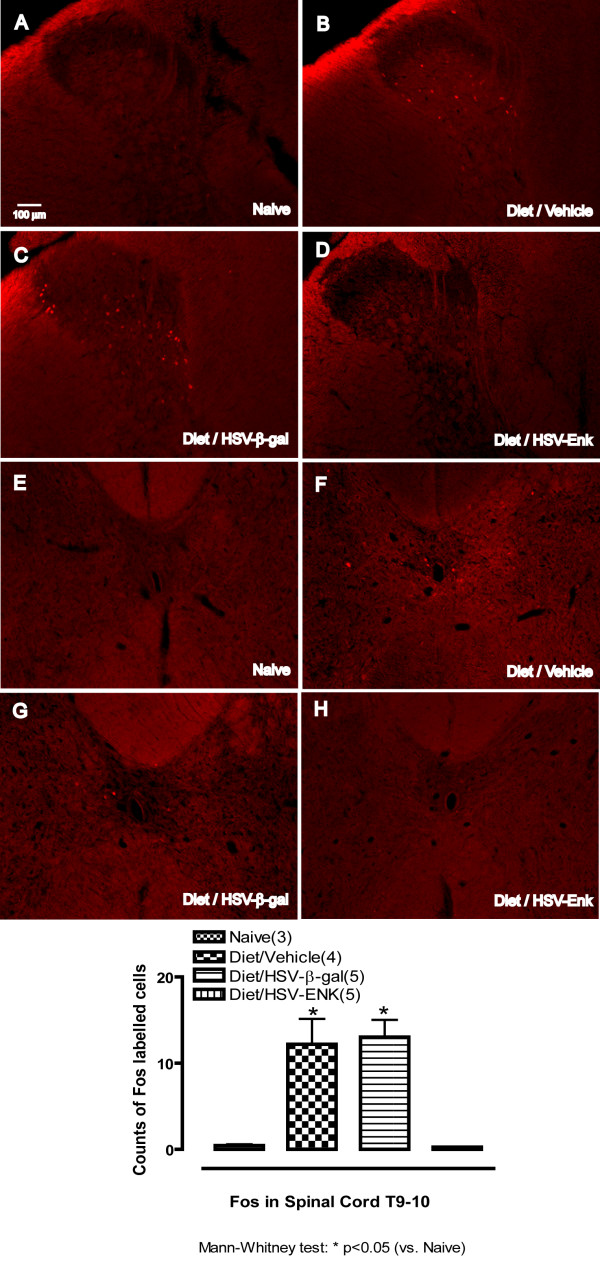
**c-Fos immunohistochemical staining in thoracic spinal cord (T9–10)**. Photomicrographs and quantification of immunohistochemical staining for c-Fos in nuclei of cells of the thoracic spinal cord (T9–10) are shown for week 10. Spinal cord dorsal horn is shown in **A-D**. Spinal cord lamina X is shown in **E-H**. Naïve rats have almost no nuclei staining for c-Fos (**A**, **E**). Significantly more nuclei staining for c-Fos are found in animals with diet-induced pancreatitis and application of vehicle (**B**, **F**) or HSV-β-gal (**C**, **G**). Few nuclei were stained for c-Fos in animals given the diet and application of HSV-ENK (**D**, **H**).

**Figure 8:**
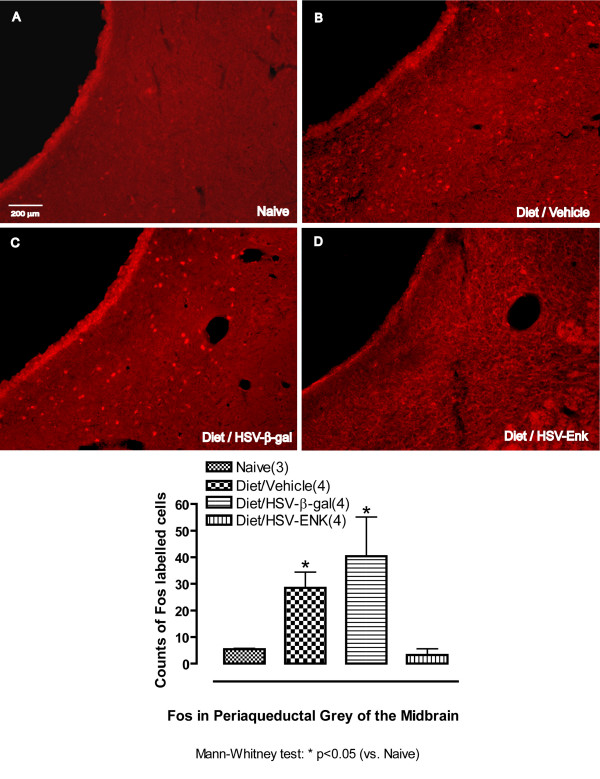
**c-Fos immunohistochemical staining in periaqueductal grey**. Photomicrographs and quantification of immunohistochemical staining for c-Fos in periaqueductal grey (PAG) (week 10). **A**. Few stained nuclei are evident in naïve rat. **B**. Significantly more c-Fos labeled nuclei are evident in animals with diet-induced pancreatitis and application of vehicle, or (**C**) in animals with diet-induced pancreatitis and application of HSV-β-gal. **D**. Little or no staining for c-Fos is evident in animals given the diet and application of HSV-ENK.

At week 10, increased numbers of cells with Fos immunostained nuclei were seen bilaterally in the deep dorsal horn and laminae X of spinal cord (T9–10) in the animals with pancreatitis treated with vehicle (Fig 7B, 7F, 12.2 ± 2.9 cells per section) or HSV-β-gal (Fig 7C, 7G, 13.0 ± 2.0 cells). In contrast, the HSV-ENK-treated animals were almost devoid of Fos staining in these areas (Fig 7D, 7H, 0.3 ± 0.1 cells), as in the naïve animals (Fig 7A, 7E, 0.4 ± 0.2 cells). In the brain stem, increased Fos expression was seen bilaterally in the nuclei of cells of the ventrolateral area of the periaqueductal grey and dorsal raphe nucleus in the animals with pancreatitis treated with vehicle (Fig 8B, 28.5 ± 6 cells) or HSV-β-gal (Fig 8C, 40.4 ± 14.8 cells). In contrast, the HSV-ENK-treated animals were almost devoid of Fos staining in neuronal nuclei in these areas (Fig 8D, 3.2 ± 2.3 cells), as in the naïve animals (Fig 8A, 5.4 ± 0.3 cells).

### Decreased RANTES expression in the pancreas of HSV-ENK-treated animals

**Figure 9:**
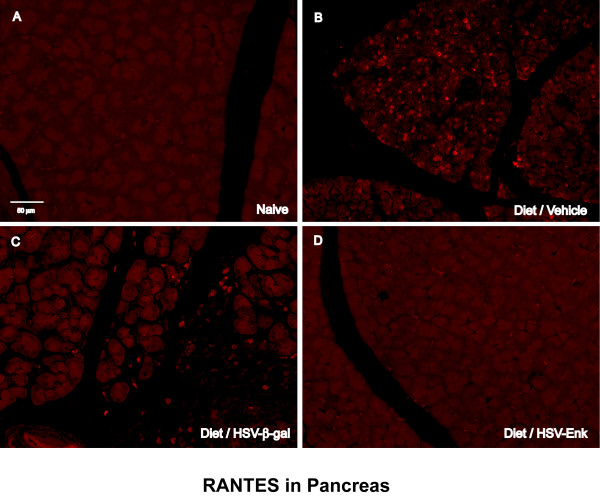
**RANTES immunohistochemical staining in pancreas**. Photomicrographs of immunohistochemical staining of RANTES in the pancreas of rats are shown for week 10. **A**. Naïve rat pancreas. **B**. Diet-induced pancreatitis and application of vehicle as control. **C**. Diet-induced pancreatitis and application of the HSV-β-gal control vector. **D**. Animals given the same diet and application of HSV-ENK. Note the increased RANTES staining in pancreata of animals with alcohol and high-fat diet induced pancreatitis treated with vehicle or HSV-β-gal applications. Little or no staining of RANTES is noted in naïve and HSV-ENK-treated animals.

Staining for the inflammatory mediator RANTES in the pancreas revealed increased expression in the vehicle- and HSV-β-gal-treated animals with pancreatitis at week 10 (Fig 9B and 9C, respectively). The RANTES staining appears primarily confined to the inflammatory cells infiltrating the pancreatic tissue. In contrast, there was negligible RANTES staining in the HSV-ENK-treated animals with pancreatitis (Fig 9D) or naïve controls (Fig 9A).

### μ-Opioid receptor expression in the pancreas

**Figure 10:**
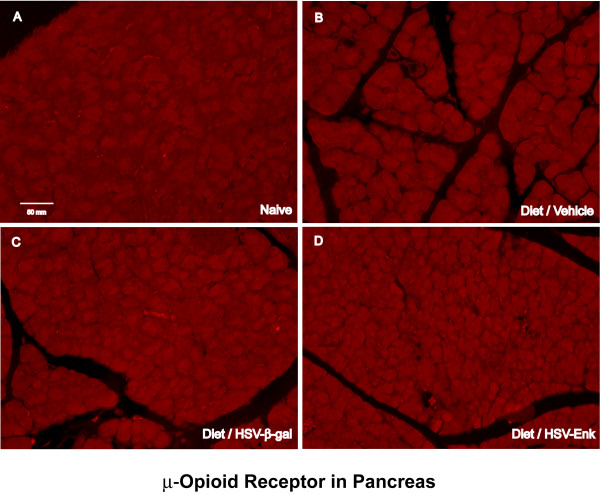
**No μ-opioid-receptor staining in pancreas at 10 weeks**. Photomicrographs of immunohistochemical staining of μ-opioid-receptor in pancreas of rats are shown at week 10. **A**. Naïve rat pancreas. **B**. Diet-induced pancreatitis and application of vehicle. **C**. Diet-induced pancreatitis and application of HSV-β-gal as HSV vector control. **D**. Pancreas from animals given diet and application of HSV-ENK. There was no significant positive staining in any of the four groups of animals.

Staining density for μ-opioid receptor in the pancreas was consistently low for all groups at week 10 (Fig 10A–D), indicating no difference in μ-opioid receptor expression at week 10. This is in contrast to high levels seen at one week after HSV-ENK treatment in an acute pancreatitis model [21]. No changes in substance P staining in the spinal cord were seen at week 10, although we have observed increased substance P receptor NK-1 expression one week after acute, chemically induced pancreatitis [22].

## Discussion

Overexpression of met-ENK observable both in spinal cord and pancreas significantly improved pancreatic inflammatory and histological outcomes, as well as reversed hotplate hypersensitivity in a chronic pancreatitis model induced with a high fat and alcohol diet in young rats. Histologic signs of chronic pancreatic inflammation evident in controls were not observed at ten weeks after HSV-ENK treatment. Diet induced pancreatitis in controls (with or without the HSV-β-gal control vector treatment) was evident as fibrosis, edema, acinar necrosis, steatosis, ductal widening, and inflammatory cell infiltration, with no hepatic or Langerhans involvement. Hotplate sensitivity apparent after 3 weeks on the diet in all animals persisted though the ten week study in controls, but was attenuated for 4–6 weeks after HSV-ENK treatment without tolerance or other adverse opiate symptomatology.

### HSV-ENK-Treated animals demonstrated normal histology and decreased inflammation

The most intriguing finding in these studies was the relative preservation of pancreatic tissue in HSV-ENK-treated rats fed the same high-fat and alcohol diet, compared to the significant inflammatory tissue damage noted in the vehicle and control vector-treated animals. The histological preservation and/or restoration of pancreatic tissue is coupled with decreased RANTES staining and inflammatory cell invasion in HSV-ENK-treated animals relative to vehicle and vector controls. Marked improvements in inflammatory cell infiltrates and decreased inflammatory mediator content (RANTES and COX-2) are seen after HSV-ENK treatment in two additional animal models in use in our laboratory (CFA arthritis and acute pancreatitis [21]). Radiographic improvement after inflammatory damage has been reported previously in arthritic animals treated with this enkephalin-encoding vector in addition to decreased periarticlar osteopenia [18]. There is abundant evidence that opioid peptides delivered by infiltrating inflammatory cells contribute to tissue enkephalin content with modest anti-inflammatory effects [23-28]. However, unlike synthetic opiates, met-enkephalin is quickly degraded by endopeptidases and is not measureable in blood. It is important to note that over-expressed met-enkephalin is delivered directly to target tissues by nociceptive afferents affected by the HSV vector [19,29]. Previous experiments, using antisera against human preproenkephalin, which does not cross-react with rodent preproenkephalin have demonstrated gene product expression in sensory neurons containing HSV protein after peripheral administration of this human preproenkephalin vector [13]. The impact of opiates delivered to the site of inflammation by the affected neuronal endings has not previously been appreciated. Delivery of significant quantities of exogenous met-enkephalin by HSV vector-driven overexpression provides a means of getting at this issue and by all appearances provides significant tissue protection. Enkephalin has been shown to reduce release of substance P from dorsal root ganglia cells in culture by blocking voltage gated Ca^++ ^channels [30], which would abrogate both nociceptive and neurogenic edema actions of substance P. We propose that it is the continuous release by the neuronal endings of significant levels of met-ENK, directly into pancreatic tissue in a physiologically relevant manner that provides additive effectiveness for tissue protection from inflammatory responses imposed by the diet. Follow-up studies will be required to assess the relevance of neuronally-released met-enkephalin to the inflammatory response.

### A protocol with accelerated alcohol associated pancreatitis

Pancreatitis is defined by the presence of inflammatory mediators and inflammatory cells in the pancreas in histological samples. The animal model provided here has histopathology consistent with severe chronic pancreatitis without mortality. The rapidly induced chronic pancreatitis, complete with fibrosis in three weeks, is likely accelerated at this developmental stage since the pancreas of young animals is ill equipped for this diet. Many previously reported pancreatitis models employing high fat and alcohol diets in older rats take much longer to develop, and pancreatitis is not consistently observed in all animals. Animals given alcohol alone as adults can take up to eight months to develop pancreatitis [1,2]. Modifications in this study from previously published models [31,32] included initiation of high fat and alcohol diet at an earlier age, incrementally increasing alcohol concentration each week in the nutritionally balance commercial diet to assure animals are able to maintain increase in body weight and good health.

The chronic pancreatitis model described here was devised specifically to test the full time course of HSV viral vector overexpression. Our accelerated diet-based chronic pancreatitis model in young animals yields consistent inflammatory pathology, does not require surgical manipulation, is cost efficient and importantly does not have the associated high mortality rate we observe with acute chemical/surgically induced pancreatitis models. Furthermore, the animals show no outward signs of discomfort or weight loss, and thus studies can be blinded through the three month time course of study. The diet induced pancreatitis was mild enough to maintain a healthy appearance in young animals for the study duration, did not produce spontaneous behavioral signs of visceral pain or altered pain thresholds as in other more severe visceral pain models, and allowed blinded behavioral testing. These data further support the observation made previously that high fat diet causes pancreatic inflammation in animals though longer times are required for adult animals [33].

### Hotplate sensitization

The pancreatitis model provided accelerated development of hotplate sensitization within three weeks in all animals tested. The hotplate test was well tolerated and allowed determination of the full time course of the HSV mediated met-enkephalin overexpression effect. This has not been possible with other pancreatitis models which either resolve within weeks or produce more severe pancreatitis with multi-organ failure possible if allowed to persist. Outright spontaneous pain behaviors were not evident in these animals, nor were results positive for other conventional pain-related behavioral tests, such as Hargreaves, von Frey and open field tests, although our preliminary tests indicate that these measures will be useful in extended studies (over three months). Although there have been few studies assessing hyperalgesia or allodynic responses in humans, both chronic pancreatitis and pancreatic cancer produce secondary thermal hyperalgesia in humans [34,35]. Chronic pancreatitis produces generalized deep hyperalgesia consistent with central sensitization [36,37]. Most pain testing in patients using mechanical and electrical stimulation, report heightened responses, VAS scores and increased descriptive words for the pain. There are at least 3 quality of life instruments that have been used in pancreatitis related pathologies. These include the SF-36, which is validated across countries, ethnicities and is non-organ specific. This test reflects the physical and mental restrictions placed on the patient secondary to generalized pain. There are also modified SF-36 instruments focused for pancreatic cancer. The standard SF-36 includes a domain for bodily pain (BP), which assesses general body pain. The normative value for BP is >85 for most studies. Pancreatic pain patients usually score around 40–50, which is significantly less, but improvement in BP is due to therapeutic interventions.

The met-enkephalin-induced analgesia suggests that spinal cord opiate receptors modulate transmission of visceral pain information that is being provided to higher centers involved in the processing affective responses to pain. Assessments of affective pain due to visceral pain are considerably more difficult in preclinical testing. The hotplate test provokes complex higher level pain induced responses (foot flick, paw licking, etc) mediated by supraspinal as well as reflexive motor commands [38]. Tests relying primarily on reflexive spinal cord and brainstem responses to stimulation of cutaneous regions of the abdomen or footpad were unaffected in this model, as was noted in a tail amputation model previously [38]. Despite measurable blood alcohol levels, open field activity testing in the San Diego apparatus revealed no differences between groups through the ten weeks of testing. This is reflective of the mild nature of the model which we have chosen for these chronic studies. It is likely that continuation of the high fat and alcohol diet would initiate referred and secondary nociceptive sensitization. Erichsen (see [39]) stated that pain from urinary bladder is sometimes referred to soles of the feet. Ness and colleagues [40] have reported sensitization as far as the knees in rats after repeated bladder distensions. The pancreas of rat is innervated bilaterally by axons from dorsal root ganglia T6-L2 (primarily T9–T13, [41]. Kuo and De Groat [42] determined that splanchnic nerve innervating pancreas (and other visceral organs) is composed of 90% unmyelinated fibers. Nociceptive foot withdrawal in response to applied heat occurs at about the same skin temperature as activation of nociceptors, thus response latency is an accurate measure of changes in nociceptive threshold produced by drug treatments [43-45]. Mechanosensitivity was not evident in this model, implying there was no recruitment of peripheral Aδ or Aβ fibers or sensitization of central neurons to this type of input. Previous studies have reported differential hyperresponsivity for mechanical without thermal sensitization [40,46-48]. An example of thermal hyperalgesia alone was shown after gene transfer for overproduction of nerve growth factor (NGF). The NGF produced thermal and mechanical hyperalgesia in the injected paw, but only thermal hyperalgesia in the uninjected paw [49]. Spinal sensitization mechanisms are likely responsible, while higher level sensitization is implied for the hotplate test. Meller and Gebhardt [50] propose that differential responses indicate that different mechanisms underlie thermal and mechanical hypersensitivity. Thermal hyperalgesia occurs with N-methyl-D-aspartate (NMDA) receptor mediated calcium-dependent production of nitric oxide, while mechanical hyperalgesia results from coactivation of alpha-amino-3-hydroxy-5-methylisoxazole-5-propionate (AMPA) and metabotropic glutamate receptor mediated cyclo-oxygenase products of arachidonic acid metabolism.

It is well known that somatic and visceral pain differ substantially in perception. In particular, a classic feature of visceral pain is referral to another part of the body [39], often following a dermatomal pattern [51]. A previous study reports thermal sensitivity of hindpaw after acute bladder inflammation (50% turpentine for 1 hr) [52] and provides review of two primary theories to explain referred pain. The axon reflex theory proposed by Sinclair, Weddell and Feindel in 1948 [53] stated that axons with collateral branches innervating both somatic and visceral targets may become sensitized sending erroneous messages to spinal cord. A second theory by Hardy and others proposes convergence at a central site that becomes an irritable focus [49,53-55]. The data presented in the present study provide support for central viscerosomatic convergence. Convergence of both visceral and somatic input has been shown onto spinal neurons located in the visceral processing region near the central canal (lamina X) [56] that projects to regions of the brain involved in processing of affective pain [57]. Thus, the hotplate test is able to distinguish spinal and supraspinal (higher order decision making) responses specific to visceral pain and analgesia in this model. Mutant enk-/- mice are rendered highly sensitive to the hotplate test while their baseline responses to other nociceptive tests, such as tail-flick and force swim, were unaffected [58]. When the knockout animals were challenged with acute pain as in the formalin test or when normal animals are treated with naloxone, nociceptive hyper-responsivity indicated a direct response to the lack of enkephalin. Konig and colleagues [58] have also speculated that opiates affect an anatomical pathway for subjective pain rather than discriminative pain. Studies in humans show that opiates do not alter baseline responses to pain (pain thresholds) but only the subjective experience of pain [59]. The present studies provide support for a role by met-enkephalin in visceral pain transmission and ultimately involvement in affective responses to pain implying that subjective pain is not entirely mediated by the many opiate receptors distributed at higher brain levels, including the limbic system.

### HSV-ENK significantly reduced behavioral sensitization without tolerance for 4 weeks

Noxious thermal stimulation has been used to assess central sensitization induced by various experimental models of clinical pain syndromes [60-63]. In this study, the hot plate test indicated hypersensitivity after three weeks on the diet. Response latencies recovered within 2 weeks to near-baseline levels in HSV-ENK-treated animals, but not in animals treated with control HSV-β-gal virus or the vehicle. The single HSV-ENK treatment provided an anti-nociceptive effect persisting for at least 4 weeks (week 5–9) without tolerance normally observed in rats with opiate therapy. This overexpression time course and efficacy profile is consistent with other HSV viral vector studies in animal models of ongoing central sensitization [13,64,65]. Thus, with further molecular manipulations, these vectors have potential for treatment of chronic pain, including visceral pain.

In a site-directed manner, pancreatic surface application selectively and effectively provides met-enkephalin to the same receptors on nerve ending receiving information from the inflamed pancreas, thus potently contributing to anti-nociception and tissue preservation/restoration. Standard pain therapies relying on higher and higher levels of circulating opiates, on the other hand, can result in intolerable side-effects in patients and rapid development of tolerance in rats within days. Differential effects are noted with mu opiate treatments, however, when an inflammatory model is used in rats [66,67]. Enkephalin administration has also been shown to attenuate morphine tolerance [68,69], though it is rapidly inactivated by endopeptidases if administered by conventional routes. The present study indicates that site specific overexpression of met-enkephalin is a suitable opiate replacement therapy or adjunct to low dose morphine, and would avoid intolerable systemic side-effects.

The gradual return of hyperalgesic responses (decreased latency thresholds) was noted between weeks 8 and 10 to the same levels seen in the control animals with pancreatitis, despite significantly high levels of met-enkephalin in the dorsal horn and pancreas. This mismatch may be either reflective of the development of tolerance by week 10 and/or diminishing synthesis of the transgene product with increasing HSV latency. Alternatively, other plastic changes may develop by ten weeks in the spinal cord of the now adult rats related to descending facilitation pathways and/or the diverse population of receptors and transcription factors known to have a role in the development of tolerance [70-72]. The literature provides few clues for the extended time course of the present study, so additional studies are warranted.

### Fos expression in animals with pancreatitis

Expression of Fos protein in the nucleus is another established indicator of cell activation, especially after noxious stimulation [73-78], including visceral pain [79-82]. With the exception of the HSV-ENK-treated animals, Fos expression was present at ten weeks in spinal cord, ventrolateral periaqueductal gray (PAG) and dorsal raphe. These regions are major central nervous system sites involved in modulation of nociception and concomitant behavioral and autonomic responses [83-85]. Noxious stimulation of viscera has been shown to evoke significant increases in Fos expression in the ventrolateral column of the PAG in acute pain models [86-89,80,86-88]. Typically, enhanced expression of Fos is reported for chronic pain models only after another acute noxious stimulation is given [89]. The presence of Fos at ten weeks in the control animals with pancreatitis was unexpected and likely related to the persisting inflammation that was providing continuous noxious stimulation of pancreatic afferents. While significant Fos expression was observed in hypersensitive control rats fed high fat and alcohol diet (vehicle and HSV-β-gal controls), little Fos was evident in animals treated with HSV-ENK, even though the hotplate sensitivity was re-established at ten weeks. It is likely that Fos protein detected by the polyclonal antibody is a chronic Fos-related antigen (FRAs; Ex. deltaFosB), a stabile protein that can persist for months which served here as a biomarker for activation only in groups with chronic ongoing pancreatic inflammation and hypersensitivity as reported previously in other inflammatory and restraint stress models [90-92]. This supports the role for met-enkephalin in long-term visceral nociceptive processing and/or autonomic control as previously speculated [93].

Interestingly, the Fos labeled nuclei in spinal cord of control animals with diet-induced pancreatitis were localized in deeper laminae of the dorsal horn rather than superficial laminae as in some pain models, i.e. cutaneous and neuropathic pain. Localization in deeper lamina has been noted previously with bladder stimulation [94]. Abbadie and Besson [95] speculated that deep laminar distribution of Fos seen in their early CFA studies might denote chronic versus acute noxious activation. We further speculate that deep tissue, whole body insults such as their CFA injections at the base of the tail or pancreatitis in the current study, activate cells deep in the dorsal horn and around lamina X, in contrast to superficial cutaneous insults which activate cells and inducing Fos in the superficial dorsal horn. The deeper distribution of activated cells is also consistent with visceral pain transmission by (1) lateral spinothalamic tract cells in laminae IV, V and VII and (2) post-synaptic dorsal column cells in laminae III and X [96].

### The role of opiate receptors in inflammation

No evidence of inflammation was seen at ten weeks in HSV-ENK-treated animals, i.e. no inflammatory cell infiltration or RANTES, despite apparent decrease in antinociceptive effect of the vector at this time point. This apparent contradiction may be related to clinical observations that analgesic effectiveness of opiates can diminish due to decrease of opiate receptors while other physiological effects of opiates are sustained. Adverse sympathetic effects occur in response to use of synthetic opioids in patients even after development of tolerance to the analgesic effects [97]. Therefore, anti-inflammatory and analgesic effects of opioids may each have uniquely dedicated mechanisms and time courses.

The improved histological findings for pancreatic tissues after HSV-ENK in the present study were coincident with decreased RANTES and COX-2 and increased met-enkephalin staining. The anti-inflammatory effects of opioids, including met-enkephalin, have been previously reported, and met-enkephalin is believed to be the major anti-inflammatory peptide of the preproenkephalin gene products (for review see [28]). Studies have shown that peripheral opioid receptor effects provided by blood borne inflammatory cells are increased in efficacy and potency during active inflammatory conditions, and a role for neuronal opioid peptides in reduction of inflammatory mediators has also been reported [21,28,98-104]. The increase in efficacy during inflammation has been shown to be true for reduction of RANTES in particular [69,105]. A previous study has shown that met-enkephalin is also protective against stress ulcers in the gastrointestinal tract of rats [106].

The literature suggests the opioid-induced immunosuppression in peripheral target cells include both opioid receptor-dependent and receptor-independent mechanisms, although the mechanisms are not fully elucidated at this time. While delta opiate receptors are abundant in the pancreas, their usual role is as an indirect mediator of the acinar secretory functions [107,108]. Delta-opioid receptor agonists such as met-enkephalin do not affect the stimulant effect of KCl on isolated pancreatic lobules or acinar cells *in vitro*, rather they decrease pancreatic enzyme secretion by inhibiting cholinergic transmission. No labeled nerves were observed in the fragile samples from the pancreas, nor were delta opiate receptor antibodies available at the time of these studies. Mu opiate receptors and met-enkephalin protein are found in the pancreas in amounts equal to the brain homogenate [109], and since they are normally involved in glucose metabolism their localization is typically in the glucagon cells of the islets of Langerhans. While mu opiate receptors were unchanged at the end of the present long term study after HSV-ENK administration, mu opiate receptors were shown to be increased at one week in our previous study in all animals with acutely inflamed pancreata [21]. The mu opiate localization evident in the pancreatic acinar cells one week after HSV-ENK is thus far unexplained. The significance of the abundance of met-enkephalin in the acini of the pancreas after HSV-ENK treatment in the absence of any remaining evidence of HSV protein, however, suggest that the met-enkephalin is of neuronal origin in agreement with previous studies with this vector or a similar one where stimulated release product was measured peripherally or stained proximally in ligated peripheral nerves [19,29].

## Conclusion

In conclusion, this study provides significant evidence for anti-inflammatory or tissue preserving effects of met-enkephalin transgene overexpression that are at least partly independent of its analgesic properties. Other significant contributory findings include demonstration of a highly effective method for site specific reversal of inflammation while using lower titers and direct tissue administration and development of an easily induced chronic pancreatic inflammation model. Sustained analgesia without tolerance is evident for at least 4 weeks after direct pancreatic application of recombinant preproenkephalin-encoding HSV vector consistent with the pre-latency expression duration for this vector. An important safety issue is addressed, i.e. there was no evidence of spread of HSV-1 centrally or peripherally other than into the appropriate level DRG. These studies provide important considerations in developing safe and effective therapeutic regimens for clinical pancreatitis and other conditions of chronic inflammatory pain. Further molecular manipulations will enable investigators to engineer viral therapies balancing greater potency, increased duration, specificity of intended effect, and safety. Re-occurrence of neurogenically initiated inflammatory bouts is common with pancreatitis, underscoring the relevance of further research toward understanding central and peripheral inflammatory pain mechanisms. While improved anti-inflammatory agents have become available in recent years, the prolonged effectiveness observed with a single dose of the neurotropic overexpression vector may in the future provide distinct advantages for some clinical applications in reducing pancreatic inflammation and pain.

## Methods

### Animals and diet

All procedures are consistent with the guidelines of the policies for Ethical Treatment of Research Animals published by the International Association for the Study of Pain, and were approved by the Animal Care and Use Committee at our institution. Young male Lewis rats (42 days old) weighing between 125–150 g (Harlan Sprague-Dawley, Houston, TX) were used for this study. Animals were kept in a 23° ± 2°C room on a 12 h light-dark cycle, three per cage until they received surgical procedures, at which time they were kept one animal per cage. Animals were divided into four groups: naïve, pancreatitis + vehicle, pancreatitis + HSV-1 with preproenkephalin cDNA (HSV-ENK), and pancreatitis + HSV-1 with β-galactosidase cDNA as the control transgene (HSV-β-gal). As part of the preliminary studies, animals were treated with HSV-ENK or HSV-β-gal or vehicle and fed a normal low soy diet (Teklab 8626, Harlan, Indianopolis). Results for these controls were similar to results for naive controls. The high-fat liquid diet used in this study (Micro-stabilized alcohol rodent liquid diet mix, LD 101A with LD 104 TestDiet, Richmond, IN) prepared fresh each day consisted of 28% fat from corn oil and safflower oil, 30.3% protein, 5% fiber, vitamins and minerals added as a dry powder to apple juice and 95% ethanol (4–6% alcohol).

### Experimental protocol

On day 1, baseline behavioral tests were obtained. The high-fat liquid diet with alcohol was initiated as follows: 4% alcohol for the 1^st ^week, 5% for 2^nd ^week, and 6% from the 3^rd ^to 10^th ^week. In the beginning of the study, a rat consumed a daily average of 50 grams of liquid diet. With normal growth, a rat consumed between 74–109 grams of liquid diet (17–25 grams dry diet) daily. The dose of alcohol was thus progressively increased from 4–6 g/rat/day. Weight gain was monitored weekly. Pancreatic enzymes (amylase and lipase), alcohol and glucose were measured in the blood at the end of the study after ten weeks on the high fat alcohol diet. Animals were observed closely every day and no evidence of alcohol intoxication (ataxic, lethargy) or stress (eye porphyrin excretion) was noted, allowing these studies to be conducted under blinded conditions. At the beginning of the 3^rd ^week and after behavioral testing, the viral vectors HSV-ENK (n = 7), HSV-β-gal (n = 7), or vehicle (n = 6) were applied to the pancreatic surface. Behavioral activities were monitored on the 1^st ^day of each week for all rats thereafter until they were sacrificed at the end of the 10^th ^week. Pancreas, liver, duodenum, bladder and colon were obtained before perfusion and immerse fixed for tissue histology and immunohistochemistry. Spinal cords (T7–12 and C6), dorsal root ganglia (DRG, T7–12 and C6) and brains were obtained for immunohistochemistry after transcardiac perfusion with buffered saline (50 ml) and fresh phosphate buffered paraformaldehyde (4%).

### Behavioral testing

The development of thermal hypersensitivity was measured by the hot plate test [110,111] in an Analgesiometer apparatus (Columbus Instruments). Rats were gently placed on a hot-plate with its anodized aluminum surface set to 50°C by an observer blinded to the treatment group. The response latency to nociceptive behaviors of shaking or licking paws, or jumping from the hot plate was recorded and animals removed immediately from the hotplate. A cut-off time point of 20 s was used to diminish the potential of thermal injury to the rats. Three trials separated by over ten minutes were averaged for each data point. Repeated testing with naive Lewis rats did not decrease with repeated testing or over time as we have seen (and others report) for Sprague-Dawley rats. Alcohol was removed from the test diet the evening prior to the behavioral testing to minimize potential interference with reflexive and motor testing. Pilot studies determined that similar young animals fed on high fat diets (28%) alone or alcohol ingestion with normal fat diets (10%) plus alcohol did not develop nociceptive behaviors and were comparable to animals fed a standard 10% fat diet. Overnight withdrawal of alcohol from animals receiving normal diets did not change nociceptive behaviors relative to alcohol naïve animals (not shown). This indicates that the hotplate sensitivity was not an effect of the alcohol withdrawal.

### HSV-based viral vectors

The HSV-1 virus vector HSV-ENK was constructed by insertion of an expression cassette containing the strong constitutive human cytomegalovirus promoter (hCMV), an SV-40 intron, the human cDNA encoding preproenkephalin and an SV-40 polyadenylation site into a shuttle plasmid containing flanking HSV DNA from the thymidine kinase region (SnaB I insertion site). The linearized shuttle plasmid and Pac I-digested DNA from virus DPZ was co-transfected into the complementing 7B cell line and recombinants selected by limiting dilution as described [112]. This virus is similar to another preproenkephalin-encoding virus reported previously to attenuate formalin-induced nociception and potentiate benzodiazepine anxiolysis when injected into the central nucleus of the amygdala [113,114]. Generation of the control virus encoding β-galactosidase under control of the hCMV promoter (named DZ or SHZ.1) has been described [115]. These recombinant viral vectors are replication-defective, created using the KOS strain of HSV with both copies of the ICP4-coding region (IE3 gene) deleted [116]. This viral vector has been used in several previous studies [13,19,65].

### Micropunctate application of viral vector to the pancreatic surface

Rats were anesthetized with sodium pentobarbital (50 mg/kg ip) and underwent midline surgical laparotomy to expose the pancreatic surface. Five microliters of treatment media suspension contained 2 × 10^6^plaque-forming-units (pfu) of the HSV-1 viral vector and was diluted (1:10) in vehicle media (Dulbecco's Modified Eagle Medium, DMEM, with 1% glycerol) from stock. This dilution was determined in preliminary studies. Treatment media was applied onto the surface of the pancreas below the attached peritoneum using a 1/2 cc U-100 insulin syringe (28 G 1/2). Two to three areas void of pancreatic duct and blood vessels were selected for HSV vector application by superficial punctate injection. After confirming that there was no bleeding from the pancreas or in the abdominal cavity, the abdominal muscle and skin were sutured. Rats were monitored closely during recovery from anesthesia and returned to the animal facility.

### Histopathology of the pancreas

On the day of sacrifice at the end of ten weeks, rats were deeply anesthetized with sodium pentobarbital (70 mg/kg). The pancreas was collected from each rat before the perfusion procedure, and washed with phosphate-buffered saline (PBS, pH 0.1 M) several times to remove blood elements, followed by immersion fixation in 4% paraformaldehyde for 1 to 2 days. Three fixed pancreata from each group were transferred to a container with 70% alcohol and sent to the UTMB histology core for paraffin embedding, sectioning and hematoxylin/eosin (HE) staining. As tissue controls, liver, bladder and colon were also processed and stained with H&E in the same manner as described above. The tissues were examined and photographed with a microscope (Nikon Eclipse E1000) equipped with a digital camera system (CoolSnap ES Monochrome, Photometrics, Roper Scientific Inc., Tucson, AZ) for histopathologic analysis.

### Immunohistochemical methods

For immunohistochemical analyses, the deeply anesthetized animals were perfused transcardially with 50 ml of heparinized saline at 37° followed by 500 ml of cold (4°) 4% paraformaldehyde solution in 0.1 M phosphate buffer (PB; pH 7.4). The brains, spinal cords, and DRG were post-fixed using the same fixative at room temperature for 4 hours before cryoprotection in 0.1 M phosphate buffered 30% sucrose solution overnight and embedding in OCT compound. Spinal cords and brainstems were cut on a sliding microtome (30 μm) and immunostained free floating in simultaneous experimental groups. Pancreata (n = 4 per group) and DRG sections were cut (10 μm) on a cryostat and placed directly on glass slides for immunostaining. The sections were rinsed (6×) in 0.1 M PBS, first incubated in 5% blocking serum at room temperature for 40 min and then, for 24 h in primary antibodies/1% NGS containing Triton X-100. Primary antibodies used in immunohistochemistry were titrated and used as follows: anti-met-enkephalin (1:1000; BioMol, Plymouth Meeting, PA), anti-HSV-1(1:500; DakoCytomation, Glostrup, Denmark), anti-β-gal (1:1000; Abcam, Cambridge, MA), anti-FOS (1:3000; Calbiochem La Jolla, CA), anti-RANTES (Regulated on Activation, Normal T-cell Expressed and Secreted;1:300; R&D System, Minneapolis, MN), anti-μ-opioid receptor (1:4000; Chemicon, Temecula, CA). Sections were washed three times in PBS and then incubated with goat anti-rabbit secondary antibody conjugated with Alexa Fluor 586 (1:1000, Molecular Probes, Eugene, OR) for 1 h at room temperature. After rinsing in PBS 3 times, sections were mounted onto gelatin-coated slides, air-dried, and coverslipped with Microscope Cover Glass (Fisher, St Louis, Mo). Specificity of immunostaining by each primary antibody was confirmed by negative staining when (1) the primary antibody was deleted or (2) normal rabbit serum was used as a neutral antibody. All sections from the different animal groups were processed at the same time with the same solutions for each tissue type.

### Quantification of staining density

For immunohistochemical quantification, data were collected using a computer-based image capture and analysis system (Meta-Vue, MetaMorph Imaging System, Molecular Devices Corp, Downington, PA) linked to a fluorescence microscope (Nikon Eclipse E1000, Lewisville, TX) via a digital camera (CoolSnap ES Monochrome, Photometrics, Roper Scientific Inc., Tucson, AZ). Under the same conditions, stained sections were observed, photomicrographs taken and stain densities quantified. The average intensity of staining in the 5 to 6 best representative sections from each animal were measured to control for artifacts in the tissue.

### Statistical analysis

Data were expressed as the average values ± standard error of the mean. Statistical analyses were performed using Prism software (Prism Software Corporation, Irvine, CA). Overall comparison with Kruskal-Wallis analysis of variance was used for the nociceptive behavioral data. Post-hoc comparisons to naïve animals were done with the Mann-Whitney U test among the HSV-ENK-, vehicle- and HSV-β-gal-treated animals fed the high fat and alcohol diet. Immunochemical density data have arbitrary units so the Kruskal-Wallis overall comparison was used. Post-hoc comparisons to naïve animals were done with the Mann-Whitney U test among the HSV-ENK-, vehicle- and HSV-β-gal-treated animals fed the high fat and alcohol diet. A p value ≤ 0.05 was considered significant for all comparisons.

## Competing interests

The author(s) declare that they have no competing interests.

## Authors' contributions

HY organized the data and prepared the plates and manuscript draft. TM injected the transgene and provided virology expertise. RC provided critical animal care and performed the behavioral studies. YL provided surgical expertise, immunostaining and data analysis. YR performed blinded data collection. SW provided the transgene produced in his laboratory. DY and KW conceived the study and participated in study design. All authors read and approved the final manuscript finalized by KW.

## Supplementary Material

Additional file 1 **No evidence of human HSV-1 protein in pancreas at week 10**. There was no evidence of HSV-1 staining in pancreas of animals from any of the groups at week 10. An antibody against human HSV-1 protein was used for the following groups: **A**. Naïve animals. **B**. Animals with alcohol and high-fat diet induced pancreatitis given vehicle or **C**. Animals with alcohol and high-fat diet induced pancreatitis given HSV-β-gal **D**. Animals fed the diet and given HSV-ENK treatment. Click here for file

Additional file 2 **No evidence of human HSV-1 protein in liver at week 10**. There was no HSV-1 immunohistochemical staining in the liver at week 10 in any of the animals. An antibody against human HSV-1 protein was used in the following groups: **A**. Naïve animals. **B**. Animals with alcohol and high-fat diet induced pancreatitis receiving vehicle. **C**. Animals with alcohol and high-fat diet induced pancreatitis receiving HSV-β-gal. **D**. Animals fed the diet receiving the HSV-ENK treatment. Click here for file
