# Supplementary figures and images for: Enkephalin-encoding herpes simplex virus-1 decreases inflammation and hotplate sensitivity in a chronic pancreatitis model
Source: Mol Pain. 2008 Feb 28;4:8. doi: 10.1186/1744-8069-4-8 (PMC2292157; doi:10.1186/1744-8069-4-8)

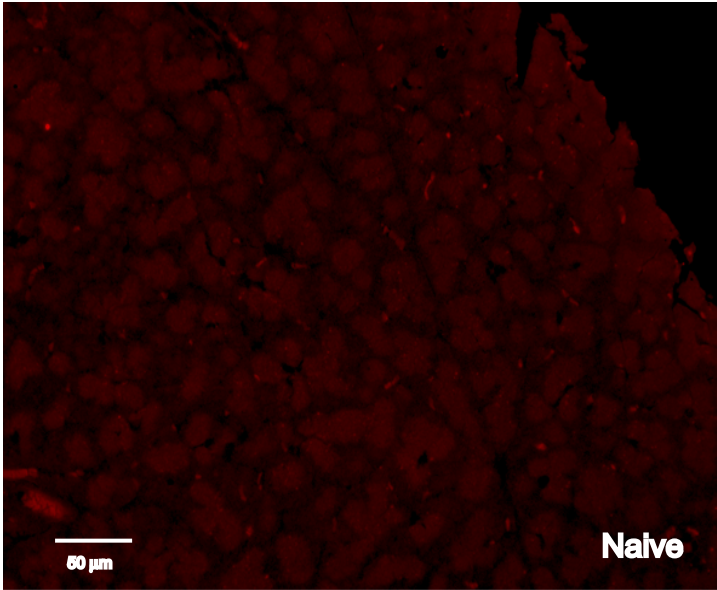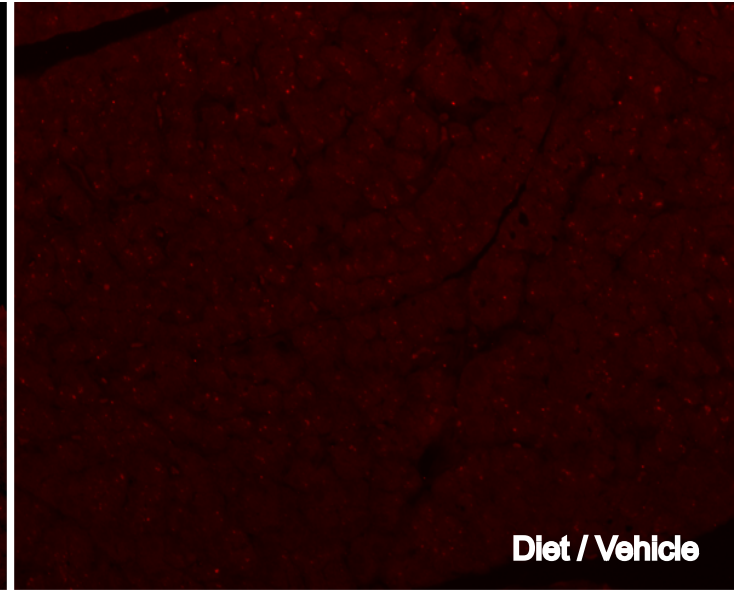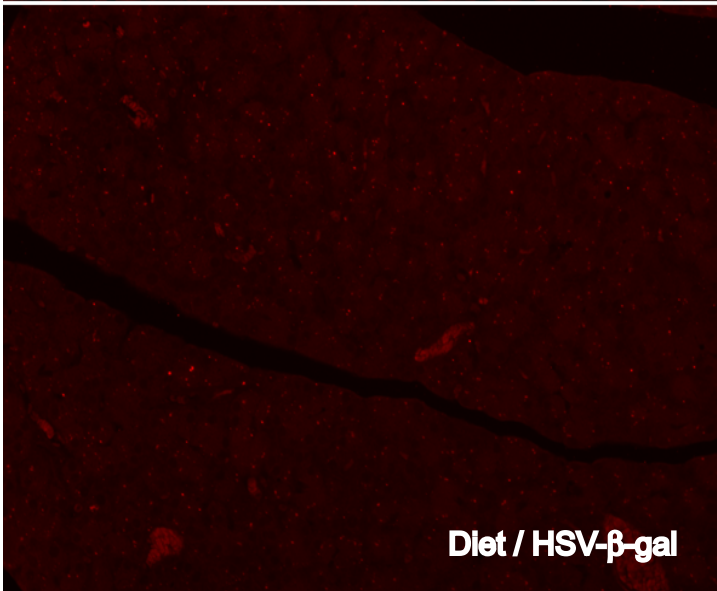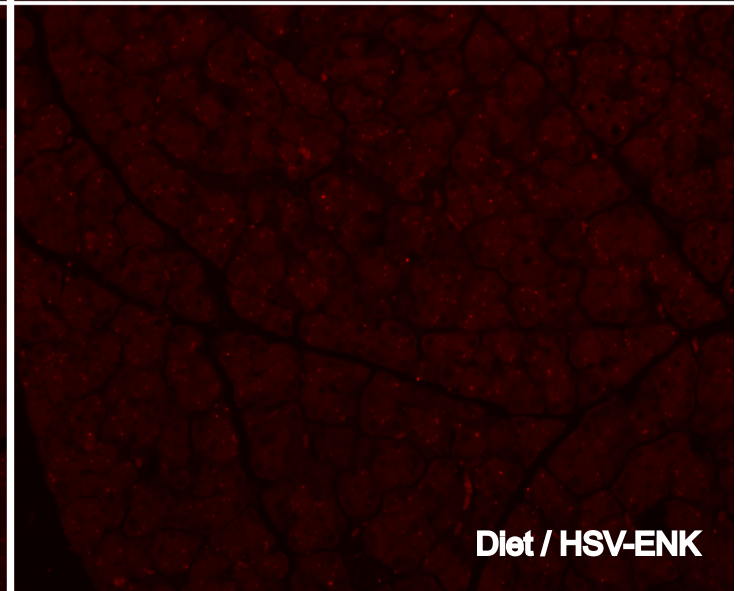

## HSV-1 in Pancreas

Supplement: Additional file 1 — No evidence of human HSV-1 protein in pancreas at week 10. There was no evidence of HSV-1 staining in pancreas of animals from any of the groups at week 10. An antibody against human HSV-1 protein was used for the following groups: A. Naïve animals. B. Animals with alcohol and high-fat diet induced pancreatitis given vehicle or C. Animals with alcohol and high-fat diet induced pancreatitis given HSV-β-gal D. Animals fed the diet and given HSV-ENK treatment. [file 1744-8069-4-8-S1.pdf]

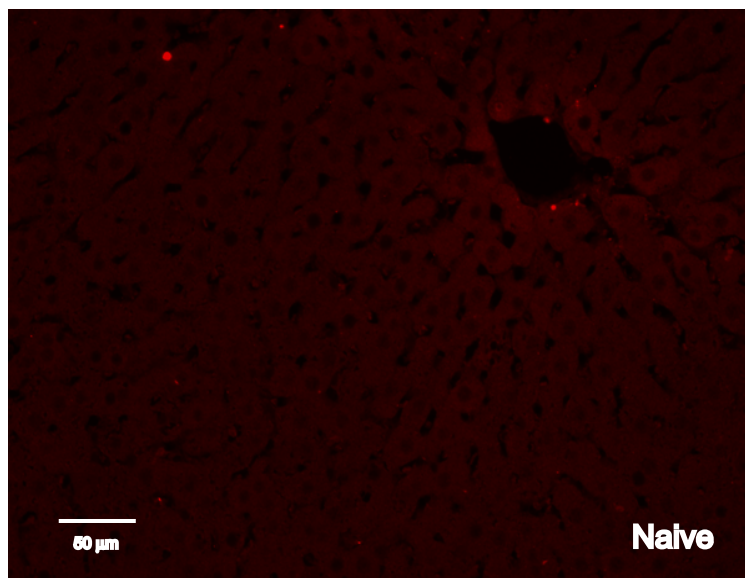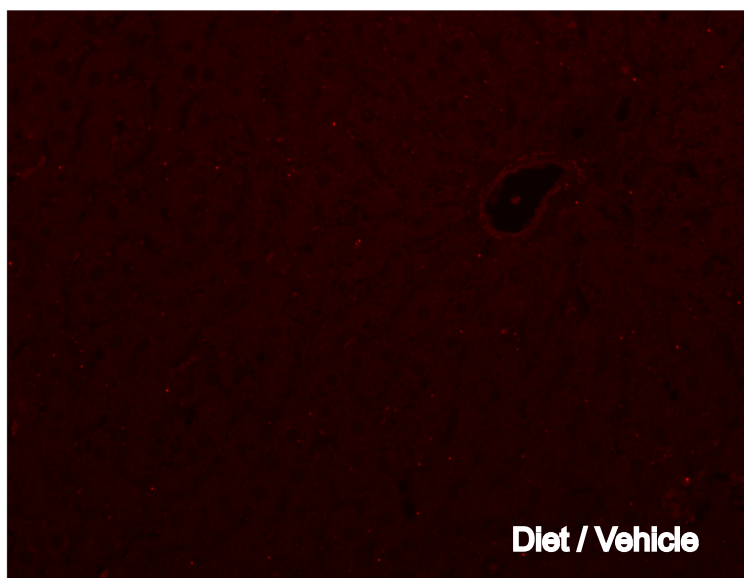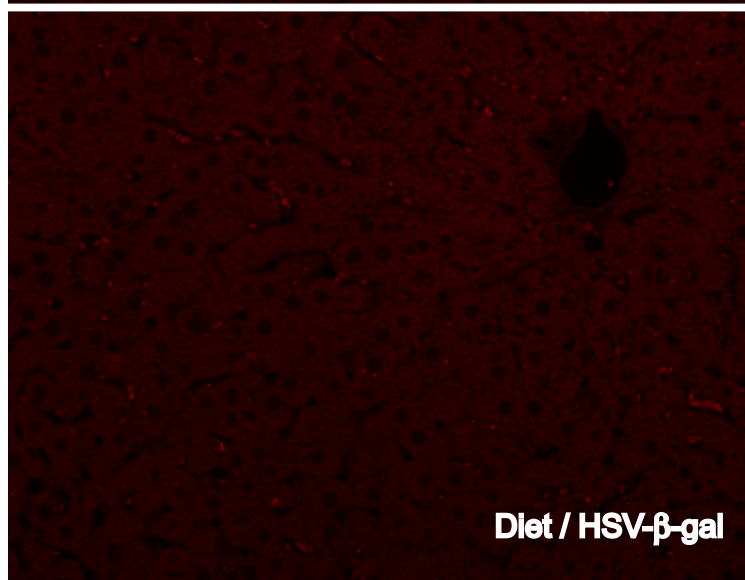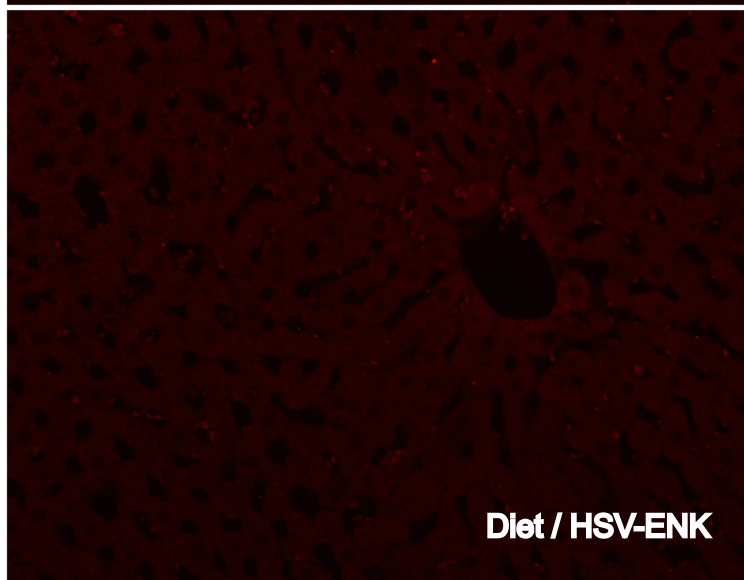

## HSV-1 in Liver

Supplement: Additional file 2 — No evidence of human HSV-1 protein in liver at week 10. There was no HSV-1 immunohistochemical staining in the liver at week 10 in any of the animals. An antibody against human HSV-1 protein was used in the following groups: A. Naïve animals. B. Animals with alcohol and high-fat diet induced pancreatitis receiving vehicle. C. Animals with alcohol and high-fat diet induced pancreatitis receiving HSV-β-gal. D. Animals fed the diet receiving the HSV-ENK treatment. [file 1744-8069-4-8-S2.pdf]
